# Attitudes toward the SARS-CoV-2 and Influenza Vaccination in the Metropolitan Cities of Bologna and Palermo, Italy

**DOI:** 10.3390/vaccines9101200

**Published:** 2021-10-18

**Authors:** Marco Montalti, Zeno Di Valerio, Flavia Rallo, Lorena Squillace, Claudio Costantino, Francesco Tomasello, Giulia Letizia Mauro, Michela Stillo, Paola Perrone, Davide Resi, Davide Gori, Francesco Vitale, Maria Pia Fantini

**Affiliations:** 1 Unit of Hygiene, Department of Biomedical and Neuromotor Sciences, Public Health and Medical Statistics, University of Bologna, 40126 Bologna, Italy; marco.montalti7@studio.unibo.it (M.M.); zeno.divalerio@studio.unibo.it (Z.D.V.); davide.gori4@unibo.it (D.G.); mariapia.fantini@unibo.it (M.P.F.); 2 Department of Public Health, Bologna Local Health Authority, 40124 Bologna, Italy; lorena.squillace@ausl.bologna.it (L.S.); michela.stillo@ausl.bologna.it (M.S.); paola.perrone@ausl.bologna.it (P.P.); davide.resi@ausl.bologna.it (D.R.); 3 Department of Health Promotion Sciences, Maternal and Infant Care, Internal Medicine and Medical Specialties (PROMISE) “G. D’Alessandro”, University of Palermo, 90127 Palermo, Italy; claudio.costantino01@unipa.it (C.C.); francescotomasello111@gmail.com (F.T.); francesco.vitale@unipa.it (F.V.); 4 Department of Surgical, Oncological, and Stomatological Sciences, University of Palermo, 90127 Palermo, Italy; giulia.letiziamauro@unipa.it

**Keywords:** vaccine hesitancy, vaccination, COVID-19, SARS-CoV-2, influenza, survey, Italy

## Abstract

Vaccine hesitancy (VH) is known to play a relevant role in thwarting the efforts toward reaching satisfactory influenza vaccination coverage, and has caused similar difficulties during the COVID-19 pandemic. This study aims to describe the phenomenon and produce insights on the reasons behind VH. A survey was administered between December 2020 and February 2021 to adults living in the cities of Bologna and Palermo. Of the 443 subjects enrolled, 47.3% were likely to get the influenza vaccination, while 75.6% were willing to receive the COVID-19 vaccination. The most frequent determinants that motivated the willingness to get the COVID-19 vaccine were trust in the safety of vaccines and belief that the vaccine is an effective tool. As for people’s unwillingness to be vaccinated, being exposed to information that produced doubts about the vaccine and lack of trust in a newly developed vaccine were the most frequently involved determinants. Statistically significant positive associations were found between the willingness to be vaccinated and postgraduate education and the propensity towards influenza vaccination. A negative association with being over 40 years old and of female gender was also found. These results might have an impact in better understanding individual reasons behind VH, identifying which categories are more exposed to it and which strategies should be implemented.

## 1. Introduction

Italy has been one of the countries most heavily hit by the pandemic. Since 21 February 2020, when the first SARS-CoV-2 infection was detected, the National Health Service has faced increasing pressure, with 4,700,316 positive cases and 131,301 COVID-19 deaths as of 13 October 2021 [1].

In Italy, public health management is shared between the state and twenty regions, which are responsible for the organization and administration of health services. The regions are autonomous territorial entities that differ in demographics, economic development, infrastructure, and even health spending, with a clear north-south divide [2]. The Emilia-Romagna region (northern Italy) and the Sicily region (southern Italy) faced enormous challenges in the context of the COVID-19 pandemic, with 387,099 and 240,535 positive cases reported to date, respectively, and it was evident in both cases that the regional capitals (Bologna and Palermo) were the territories most affected by the pandemic [3,4].

On 27 December 2020, the government and regions promoted a COVID-19 vaccination campaign by first immunizing HCWs and all personnel working in healthcare settings. From February onward, the campaign targeted elderly people over 80 and people with comorbidities and gradually expanded to increasingly younger age groups [5].

Even in the context of the COVID-19 vaccination campaign, vaccine hesitancy (VH), listed by the World Health Organization (WHO) as one of the ten global health threats of 2019, [6] and defined as “delayed acceptance or refusal of vaccination despite the availability of vaccine services” [7], played a key role. VH varies in form and intensity depending on where and when it occurs and which vaccine is affected, as confirmed by several studies and reviews on the topic [8,9,10].

Another key issue addressed within the WHO Global Health Threats report concerns the need to achieve adequate influenza vaccination coverage [6], a goal that has been viewed now more than ever as a public health priority [11].

In Italy, over the last five years, vaccination coverage levels against influenza in the over-65 population show a progressive increase, starting from 49.9% in 2015/2016 to 65.3% during the last year [12]. However, the goals are still distant, considering that in order to significantly reduce morbidity, complications and mortality due to influenza, high vaccination coverage must be achieved in the target population groups, particularly in the elderly (over 65 years of age) and in high-risk individuals of all ages [12].

Understanding the factors limiting a successful reaching of population vaccination coverage for these vaccines is crucial to suggesting implementation strategies for the dual epidemics’ vaccination campaigns.

The aim of this study was to investigate the potential determinants of both COVID-19 and influenza VH in the metropolitan cities of Bologna (Northern Italy) and Palermo (Southern Italy), and to investigate the previous influenza VH as a possible determinant of COVID-19 VH.

## 2. Materials and Methods

An in-person survey was self-administered to people older than 18 years of age living in the metropolitan cities of Bologna and Palermo [13] that were recruited between December 2020 and February 2021. People were recruited in five pharmacies located in the city of Palermo and while they were waiting to undergo a screening swab by the local health authority in Bologna. Adherence to the study was voluntary and data were collected anonymously.

### 2.1. Questionnaire

Socio-demographic variables were collected. VH contextual, individual/group and vaccine-specific determinants, chosen according to those identified by the WHO Sage Group [14], were investigated. The same vaccine hesitancy variables were transformed into affirmative outcomes to investigate vaccination propensity factors. Lastly, participants were also asked whether they had received the influenza vaccination in the year 2020 and if their decision had been influenced by the SARS-CoV-2 pandemic. A complete English version of the survey instrument can be found in the supplementary materials.

### 2.2. Statistical Analysis

Variables were described as absolute and relative frequencies. Univariate analysis was performed using the χ2 test for all categorical variables. Independent variables for which the *p* value was 0.25 or less in the univariate analysis were included in the multivariate logistic regression models. A two-sided *p* value of 0.05 or less was considered an indicator of a statistically significant difference. The following independent variables were included if they met the mentioned criteria: age (<40 = 0; ≥40 = 1), city (Palermo = 0; Bologna = 1), gender (male = 0; female = 1), level of education (<High school diploma = 0; ≥High school diploma = 1), and propensity for influenza vaccination (no = 0; yes = 1). Multivariable model results are shown as odds-ratio (OR) with confidence interval (95% CI) and *p* values. The statistical analysis was performed using Stata statistical software 14 (StataCorp, College Station, TX). The Ethics Committee of the University of Bologna, Italy, approved the study protocol (Prot n. 223916, 09/10/2020).

## 3. Results

Overall, four hundred and forty-three subjects completed the questionnaire, with 217 (49.0%) having been recruited in Palermo and 226 (51.0%) in Bologna. The main sociodemographic features of the sample and the univariate analysis are reported in Table 1. Overall, 224 (50.6%) participants were under the age of 40, and 219 (49.4%) were 40 or above, while 193 (43.6%) of them were male and 250 (56.4%) female. With regard to education, 227 (51.7%) participants had a lower certification than a high school diploma, while 212 (49.3%) had at least a high school diploma. When asked if they were likely to be vaccinated against influenza, 240 (54.7%) answered negatively while 199 (47.3%) answered affirmatively. Overall, 145 (32.7%) of the respondents thought their choice to be vaccinated against influenza was influenced by the SARS-CoV-2 pandemic.

### 3.1. Willingness to Receive COVID-19 Vaccination

Among all the participants, 335 (75.6%) were willing to receive the COVID-19 vaccination, whereas 108 (24.4%) were reluctant. In Bologna the same responses were 163 (72.1%) and 63 (27.9%), respectively, while in Palermo it was172 (79.3%) and 45 (20.7%), respectively. In Table 2, reasons for a willingness to receive the-COVID-19 vaccination are reported.

Among the cohort from Bologna, for respondents willing to be vaccinated, the most frequent determinants cited as having influenced their decision were trust in the safety of vaccines (63.2%), belief that vaccination is an effective tool (44.8%), not having had any unpleasant personal experiences with previous vaccinations (35.0%), and trust in doctors and health personnel (33.7%). In the cohort from Palermo, they were: belief that vaccination is an effective tool (67.4%), not having had any unpleasant personal experiences with previous vaccinations (59.9%), and belief that the risk of contracting COVID-19 is much greater than the risks of vaccination (48.8%).

Among respondents not willing to be vaccinated in Bologna, the most frequent determinants cited as having influenced their decision were lack of trust in a newly developed vaccine (58.7%), having read information that made them doubt the vaccine (41.3%), and not wanting politics to force them to take the vaccine (25.4%). In the cohort from Palermo, they were: lack of trust in a newly developed vaccine (55.5%), having read information that made them doubt the vaccine (26.7%), and knowing of cases of people damaged by vaccines (24.4%). Reasons for a willingness to receive the COVID-19 vaccination are shown in detail for each single city in Table S1.

### 3.2. Multivariate Logistic Regression Model of Determinants for Vaccine Confidence

Multivariate analysis (Table 3) showed a statistically significant positive association between willingness to be administered the COVID-19 vaccination and ≥high school diploma (OR = 1.69, 95% CI: 1.04–2.76) and propensity for influenza vaccination (OR = 5.36, 95% CI: 3.12–9.21). A significant negative association with being 40 years old or more (OR = 0.32, 95% CI: 0.19–0.55) and female gender (OR = 0.53, 95% CI: 0.32–0.87) emerged as well.

## 4. Discussion

In this multicenter study conducted at the beginning of the COVID-19 vaccination campaign in Italy, we found a high overall vaccine acceptance rate (75.6%), in line with other studies that detected VH in the general population during the same time period (70.9%–77.3%) [10,15,16]. Vaccine confidence rates were found to be not particularly different between the two regional capitals surveyed, with 79.3% and 72.1% for Palermo and Bologna, respectively.

Our findings show that males, participants with a higher level of education and younger people (<40 years) were more likely to get a COVID-19 vaccine. As for males, a higher vaccine confidence has already been documented by several studies [17,18,19,20,21], and could be explained by a higher risk perception of COVID-19, which has been shown from the beginning of the pandemic to be particularly severe in this specific population [22]. Similarly, what was found regarding the level of education corresponds with the current scientific literature, with a higher level of education being a protective factor against VH for COVID-19 vaccines [23,24,25]. This could be due to a greater availability of tools for critical analysis of fake news, the use of more reliable sources of information, and of a greater trust in institutions.

On the other hand, our finding regarding age is particularly surprising in light of the fact that COVID-19 disease has a significant impact in terms of morbidity and mortality in older people [26,27]. This could be explained by the fact that younger people, less affected by the disease, possibly see the vaccine as the most rapid and efficient means to return to their normal lives [28]. In another study, conducted on a representative sample from the Emilia-Romagna region, we found how the relationship between age and vaccine confidence could be described with a U-shape [29], while in several studies increasing age has been shown to be a protective factor against VH [22,25,30], possibly due to a higher reliance on traditional information sources and less exposure to social media [31]. The difference we found could be explained by the particular settings in which the questionnaires were collected (pharmacies and swab-screening for SARS-CoV-2) and the period in which the study was performed (beginning of the vaccination campaign). In fact, it is well established that VH is a highly complex and context-specific phenomenon that depends on place and time [32].

When discussing VH determinants, considering the vaccine as an effective tool for oneself/one’s family/community (56.4%) proved to be the main reason for vaccine confidence in respondents who were willing to get vaccinated. Other determinants that most frequently led to vaccine confidence were found to be trust in vaccine safety (51.0% of participants who were willing to get vaccinated) and the fact that respondents had never had problems with vaccinations in the past (34.3%). Past adherence to vaccinations and, consequently, having experienced common adverse reactions and having observed vaccination safety firsthand are indeed considered to be contributing predisposing factors for vaccine confidence [33,34,35,36].

We found that the fact that the vaccines in use in the COVID-19 campaign were only newly developed (57.4%) and having read any information (internet/media/social media) that raised doubts about vaccination (35.2%) were the most common reasons for unwillingness to get the COVID-19 vaccine. This finding is in line with current literature that identifies the use of social media as a contributing factor to vaccine hesitancy [31,37,38,39]. New sources of information have certainly played a crucial role in the COVID-19 vaccination campaign, which has been littered more than ever with fake news and anti-vax contents [40,41]. This phenomenon has come to be known as an ‘infodemic’, described by the WHO as the presence of too much information, including false or misleading information, causing confusion and risky behaviour that can damage health [42].

Finally, as expected, propensity toward influenza vaccination was a significant predictor of willingness to be vaccinated against COVID-19, confirming the results of previous papers [20,43,44]. It is possible that boosting confidence in a well-known and routinely administered vaccine like the influenza vaccine could lead to an improved COVID-19 vaccine uptake as well. This indirect effect on COVID-19 mortality, severity and spread might be further amplified by a direct protective effect of influenza vaccination independent of COVID-19 vaccination, which has been proposed by recent literature [45,46,47].

Interestingly, nearly one third of respondents appeared to think the COVID-19 pandemic has had a relevant impact on their decision to get vaccinated against influenza. Although we have not investigated the final effect of this influence, the existence of the effect confirms the known large impact that widely-discussed news and real world events have on VH [48,49].

## 5. Limitations and Conclusions

Some limitations should be disclosed. First, non-randomly selected participants were enrolled during a screening program performed among parents/guardians of children aged 0–18 years (Bologna, Italy) or among customers older than 18 years of age with access to five community pharmacies located in the municipality and in the province of Palermo who voluntarily joined the study. Therefore, (a) it is not possible to calculate a survey participation rate, (b) it is not possible to indicate the confidence limits of the estimated prevalence of VH, and (c) it is not clear which subset of the population the survey participants represent. In addition, the survey was conducted at the beginning of the Italian COVID-19 vaccination campaign when the public debate on this topic was not yet politicized or polarized, so the data may not be representative of people’s true perceptions. Furthermore, although our model had acceptable goodness of fit, the set of predictors found cannot be considered exhaustive because other unknown variables may play a role in determining hesitation.

Despite these limitations, the main strengths of the study are the large cohort of enrolled subjects, the multicentricity of the survey conducted in two different regional territories, the absence of operator influence on questionnaire completion, and the homogeneous distribution of participants by age, sex, and educational level.

Results produced at different levels are needed to inform policy-makers and public health professionals in their efforts to target information and communication campaigns to counter vaccine hesitancy. Our study highlights the need to implement an accurate, informative and communicative campaign dedicated to those who are more hesitant to receive COVID- 19 vaccination (e.g., the female gender, adults, people with a low level of education or who do not usually adhere to the influenza vaccination campaign). Considering the regional contribution in Italy to the organisation of the COVID-19 vaccination campaign and to the management of the pandemic, these results may represent an important element of knowledge to reinforce the need for continuous surveys conducted at different territorial levels and times.

## Figures and Tables

**Table 1:** Main features of the sample and propensity for COVID-19 vaccination according to several socio-demographic determinants.

Feature	Total N (%)	Propensity for COVID-19 Vaccination N (%)	*p*
	443	335 (75.6)	
**Age**			**χ^2^ = 16.96, 1 df *p* < 0.001**
<40	224 (50.6)	188 (83.9)	
≥40	219 (49.4)	147 (67.1)	
**City**			χ^2^ = 3.06, 1 df *p* = 0.080
Palermo	217 (49)	172 (79.3)	
Bologna	226 (51)	163 (72.1)	
**Gender**			**χ^2^ = 6.08, 1 df *p* = 0.014**
Male	193 (43.6)	157 (81.4)	
Female	250 (56.4)	178 (71.2)	
**Level of education ^a^**			χ^2^ = 1.86, 1 df *p* = 0.172
<High School Diploma	227 (51.7)	165 (72.7)	
≥High School Diploma	212 (48.3)	166 (78.3)	
**Propensity for Influenza vaccination ^a^**			**χ^2^ = 36.01, 1 df *p* < 0.001**
No	240 (54.7)	154 (64.2)	
Yes	199 (45.3)	177 (88.9)	

^a^ Total may not be equal to “N” due to missing data.

**Table 2:** Reasons for willingness to receive COVID-19 vaccination in Bologna and Palermo. More than one answer was possible for each question, with no limit.

Question	Answer	Total N (%)
**If Yes, which determinants are mainly associated with your decision?**	I follow people or groups (political/religious/influencers) that make me trust the vaccine	17 (5.1)
	I would trust a newly developed vaccine	43 (12.8)
	People I know tend to be confident about the vaccine being released	47 (14.0)
	I do not believe the vaccine is a tool for the enrichment of lobbies/pharmaceutical companies	54 (16.1)
	I am not afraid of the pain nor the mode of administration	75 (22.4)
	I trust the government with deciding which vaccines are mandatory	75 (22.4)
	I have read information (internet/media/social media) that makes me trust the vaccine	86 (25.7)
	In the past I have never had any problems in accessing vaccinations	115 (34.3)
	I trust doctors and health personnel	133 (39.7)
	I believe that the risk of contracting COVID-19 is much greater than the risks of vaccination	138 (41.2)
	I have not had any unpleasant personal experiences with previous vaccinations	160 (47.8)
	I have trust in the safety of vaccines	171 (51.0)
	The vaccine is an effective tool for me/my family/my community	189 (56.4)
**If No, which determinants are mainly associated with your decision?**	In the past I have had problems accessing vaccinations	1 (0.9)
	I don’t trust doctors and health personnel	3 (2.8)
	I am afraid of the pain or the mode of administration	3 (2.8)
	The vaccine is a tool for the enrichment of lobbies/pharmaceutical companies	4 (3.7)
	I have had some unpleasant personal experiences with previous vaccinations	4 (3.7)
	The people I know tend to be unconfident about the vaccine being released	6 (5.6)
	I follow people or groups (political/religious/influencers) that make me doubt the vaccine	9 (8.3)
	I think COVID-19 is not threatening enough for me to risk vaccine injuries	9 (8.3)
	The vaccine is not an effective tool for me/my family/my community	10 (9.0)
	I know cases of people damaged by vaccines	21 (19.4)
	I don’t want politics to force me to take the vaccine	22 (20.4)
	I have read information (internet/media/social media) that makes me doubt the vaccine	38 (35.2)
	I wouldn’t trust a newly developed vaccine	62 (57.4)

**Table 3:** Multivariate logistic regression model of determinants for Vaccine Confidence.

Variables	OR	SE	95% CI	*p*
Log likelihood = −208.14, χ^2^ = 71.30 (5 df), *p* < 0.00001, No. of obs = 435				
**Age**				
<40	1.00 ^a^			
≥40	0.32	0.09	0.19–0.55	**<0.001**
**City**				
Palermo	1.00 ^a^			
Bologna	0.9	0.24	0.53–1.53	0.702
**Gender**				
Male	1.00 ^a^			
Female	0.53	0.13	0.32–0.87	**0.012**
**Level of Education**				
<High School Diploma	1.00 ^a^			
≥High School Diploma	1.69	0.42	1.04–2.76	**0.035**
**Propensity for Influenza vaccination**				
No	1.00 ^a^			
Yes	5.36	1.48	3.12–9.21	**<0.001**

^a^ Variable set as a benchmark.

## Supplementary Materials

The following are available online at https://www.mdpi.com/article/10.3390/vaccines9101200/s1, Survey Instrument and Table S1.
