# Supplementary material for: Attitudes toward the SARS-CoV-2 and Influenza Vaccination in the Metropolitan Cities of Bologna and Palermo, Italy
Source: Vaccines (Basel). 2021 Oct 18;9(10):1200. doi: 10.3390/vaccines9101200 (PMC8538834; doi:10.3390/vaccines9101200)
Supplement: Supplementary file 1 [file vaccines-09-01200-s001.zip › vaccines-1426089-supplementary.pdf]

Supplementary Materials

Survey Instrument

Personal information

- City
- Age
- Gender
- Educational level

COVID-19 vaccination

- **Are you willing to receive COVID-19 vaccination?**
- **If Yes, which determinants are mainly associated with your decision?**
  - I I have read information (internet/media/social media) that makes me trust the vaccine
  - I follow people or groups (political/religious/influencers) that make me trust the vaccine
  - I have trust in the safety of vaccines
  - I trust the government with deciding which vaccines are mandatory
  - In the past I have never had any problems in accessing vaccinations
  - I do not believe the vaccine is a tool for the enrichment of lobbies/pharmaceutical companies
  - I have not had any unpleasant personal experiences with previous vaccinations
  - The vaccine is an effective tool for me/my family/my community
  - I would trust a newly developed vaccine
  - I trust doctors and health personnel
  - I am not afraid of the pain nor the mode of administration
  - I believe that the risk of contracting COVID-19 is much greater than the risks of vaccination
  - People I know tend to be confident about the vaccine being released
- **If No, which determinants are mainly associated with your decision?**
  - I have read information (internet/media/social media) that makes me doubt the vaccine
  - I follow people or groups (political/religious/influencers) that make me doubt the vaccine
  - I know cases of people damaged by vaccines
  - I don't want politics to force me to take the vaccine
  - In the past I have had problems accessing vaccinations
  - The vaccine is a tool for the enrichment of lobbies/pharmaceutical companies
  - I have had some unpleasant personal experiences with previous vaccinations
  - The vaccine is not an effective tool for me/my family/my community
  - I wouldn't trust a newly developed vaccine
  - I don't trust doctors and health personnel
  - I am afraid of the pain or the mode of administration
  - I think COVID-19 is not threatening enough for me to risk vaccine injuries
  - The people I know tend to be unconfident about the vaccine being released

Influenza vaccination

- Are you inclined or have you already been vaccinated against influenza during the 2020/2021 season?
- Is or was your choice to vaccinate against influenza affected by the SARS-CoV-2 pandemic?

Table S1. Willingness to receive COVID-19 vaccination in Bologna (n=226) and Palermo (n=217).

Question	Answer	Bologna N(%)	Palermo N(%)	Total N(%)
**Are you willing to receive COVID-19 vaccination?**	No	63 (27.9)	45 (20.7)	108 (24.4)
	Yes	163 (72.1)	172 (79.3)	335 (75.6)
**If Yes, which determinants are mainly associated with your decision?** [a]	I have read information (internet/media/social media) that makes me trust the vaccine	46 (28.2)	40 (23.3)	86 (25.7)
	I follow people or groups (political/religious/influencers) that make me trust the vaccine	10 (6.1)	7 (4.1)	17 (5.1)
	I have trust in the safety of vaccines	103 (63.2)	68 (39.5)	171 (51.0)
	I trust the government with deciding which vaccines are mandatory	17 (10.4)	58 (33.7)	75 (22.4)
	In the past I have never had any problems in accessing vaccinations	54 (33.1)	61 (35.5)	115 (34.3)
	I do not believe the vaccine is a tool for the enrichment of lobbies/pharmaceutical companies	27 (16.6)	27 (15.7)	54 (16.1)
	I have not had any unpleasant personal experiences with previous vaccinations	57 (35.0)	103 (59.9)	160 (47.8)
	The vaccine is an effective tool for me/my family/my community	73 (44.8)	116 (67.4)	189 (56.4)
	I would trust a newly developed vaccine	15 (9.2)	28 (16.3)	43 (12.8)
	I trust doctors and health personnel	55 (33.7)	78 (45.3)	133 (39.7)
	I am not afraid of the pain nor the mode of administration	28 (17.2)	47 (27.3)	75 (22.4)
	I believe that the risk of contracting COVID-19 is much greater than the risks of vaccination	54 (33.1)	84 (48.8)	138 (41.2)
	People I know tend to be confident about the vaccine being released	19 (11.7)	28 (16.3)	47 (14.0)
**If No, which determinants are mainly associated with your decision?** [a]	I have read information (internet/media/social media) that makes me doubt the vaccine	26 (41.3)	12 (26.7)	38 (35.2)
	I follow people or groups (political/religious/influencers) that make me doubt the vaccine	7 (11.1)	2 (4.4)	9 (8.3)
	I know cases of people damaged by vaccines	10 (15.9)	11 (24.4)	21 (19.4)
	I don't want politics to force me to take the vaccine	16 (25.4)	6 (13.2)	22 (20.4)
	In the past I have had problems accessing vaccinations	1 (1.6)	0 (0.0)	1 (0.9)
	The vaccine is a tool for the enrichment of lobbies/pharmaceutical companies	3 (4.8)	1 (2.2)	4 (3.7)
	I have had some unpleasant personal experiences with previous vaccinations	3 (4.8)	1 (2.2)	4 (3.7)
	The vaccine is not an effective tool for me/my family/my community	5 (7.9)	5 (11.1)	10 (9.0)
	I wouldn't trust a newly developed vaccine	37 (58.7)	25 (55.5)	62 (57.4)
	I don't trust doctors and health personnel	2 (3.2)	1 (2.2)	3 (2.8)
	I am afraid of the pain or the mode of administration	1 (1.6)	2 (4.4)	3 (2.8)
	I think COVID-19 is not threatening enough for me to risk vaccine injuries	9 (14.3)	0 (0.0)	9 (8.3)
	The people I know tend to be unconfident about the vaccine being released	1 (1.6)	5 (11.1)	6 (5.6)
